# Coupled Flip-Flop Model for REM Sleep Regulation in the Rat

**DOI:** 10.1371/journal.pone.0094481

**Published:** 2014-04-10

**Authors:** Justin R. Dunmyre, George A. Mashour, Victoria Booth

**Affiliations:** 1 Department of Mathematics, University of Michigan, Ann Arbor, Michigan, United States of America; 2 Department of Anesthesiology, University of Michigan Medical School, Ann Arbor, Michigan, United States of America; 3 Neuroscience Graduate Program, University of Michigan, Ann Arbor, Michigan, United States of America; 4 Department of Mathematics, Frostburg State University, Frostburg, Maryland, United States of America; Georgia State University, United States of America

## Abstract

Recent experimental studies investigating the neuronal regulation of rapid eye movement (REM) sleep have identified mutually inhibitory synaptic projections among REM sleep-promoting (REM-on) and REM sleep-inhibiting (REM-off) neuronal populations that act to maintain the REM sleep state and control its onset and offset. The control mechanism of mutually inhibitory synaptic interactions mirrors the proposed flip-flop switch for sleep-wake regulation consisting of mutually inhibitory synaptic projections between wake- and sleep-promoting neuronal populations. While a number of synaptic projections have been identified between these REM-on/REM-off populations and wake/sleep-promoting populations, the specific interactions that govern behavioral state transitions have not been completely determined. Using a minimal mathematical model, we investigated behavioral state transition dynamics dictated by a system of coupled flip-flops, one to control transitions between wake and sleep states, and another to control transitions into and out of REM sleep. The model describes the neurotransmitter-mediated inhibitory interactions between a wake- and sleep-promoting population, and between a REM-on and REM-off population. We proposed interactions between the wake/sleep and REM-on/REM-off flip-flops to replicate the behavioral state statistics and probabilities of behavioral state transitions measured from experimental recordings of rat sleep under ad libitum conditions and after 24 h of REM sleep deprivation. Reliable transitions from REM sleep to wake, as dictated by the data, indicated the necessity of an excitatory projection from the REM-on population to the wake-promoting population. To replicate the increase in REM-wake-REM transitions observed after 24 h REM sleep deprivation required that this excitatory projection promote transient activation of the wake-promoting population. Obtaining the reliable wake-nonREM sleep transitions observed in the data required that activity of the wake-promoting population modulated the interaction between the REM-on and REM-off populations. This analysis suggests neuronal processes to be targeted in further experimental studies of the regulatory mechanisms of REM sleep.

## Introduction

Theories on the neuronal control for rapid eye movement (REM) sleep have been dominated by the cholinergic hypothesis (see [1] for review). Based on a wealth of experimental evidence collected since the identification of REM sleep in the 1950s [2], the cholinergic hypothesis posits that the REM sleep state is initiated and maintained by the activity of cholinergic neurons in areas of the pons, including the laterodorsal and pedunculopontine tegmental nuclei (LDT/PPT). This hypothesis is synthesized in the reciprocal interaction model [3], [4] for REM sleep in which regular transitions between REM and nonREM (NREM) sleep are generated by excitatory and inhibitory synaptic projections between the cholinergic REM-promoting (REM-on) LDT/PPT and monoaminergic, REM-suppressing (REM-off) neuronal populations including the locus coeruleus (LC) and the dorsal raphe (DR).

Recent experimental results have challenged the cholinergic hypothesis for REM sleep control with the identification of several additional neuronal groups with REM-on and REM-off activity profiles [5]–[7]. In the rat, REM-on groups include the sublaterodorsal tegmental nucleus (SLD), portions of the ventrolateral periaqueductal gray matter (vlPAG), areas of the lateral hypothalamus (LH) and the dorsal paragigantocellular nucleus (DPGi). Neuronal groups with REM-off activity profiles include other portions of the vlPAG and the dorsal part of the deep mesencephalic nucleus (dDPME). Based on the identification of these REM-associated neuronal groups and their synaptic interactions, alternative theories for the neuronal control of REM sleep have been proposed wherein GABA is the primary neurotransmitter and mutually inhibitory synaptic interactions govern activity of these groups and thus transitions of REM sleep [5], [7]. However, debate continues as to which of these GABAergic populations are integrally responsible for generating transitions into and out of REM sleep. For example, Lu and colleagues [5] propose that REM regulation is controlled by a core REM-on/REM-off flip-flop switch composed of mutually inhibitory (GABAergic) synaptic projections between REM-off neurons in the vlPAG and adjacent lateral pontine tegmentum (LPT), and REM-on neurons in the SLD. On the other hand, Luppi and colleagues [8] propose that REM sleep transitions are controlled by a more distributed network of inhibitory projections among REM-off neurons in the vlPAG and dDPME, and REM-on neurons in the vlPAG, LH and DPGi.

Another factor influencing REM sleep generation is the REM sleep homeostatic drive. Multiple studies have provided evidence that REM sleep is homeostatically regulated independently from NREM sleep [9], [10]. For example, increases in attempts to transition into REM sleep have been observed during REM sleep deprivation studies in which these transitions are prevented, and REM sleep rebound reliably occurs during recovery sleep from periods of REM sleep deprivation as short as 2 hours [10]. Additionally, anesthetics have differential effects in terms of satisfying the needs of NREM and REM sleep following sleep deprivation, suggesting separate mechanisms for NREM and REM sleep homeostasis. In particular, anesthesia induced by the inhaled anesthetic sevoflurane following total sleep deprivation eliminated homeostatic increases in NREM sleep but not in REM sleep [11]. The physiological substrate dictating REM sleep homeostasis has not been identified. Recent studies have suggested that melanin-concentrating hormone (MCH) [12] or the satiety molecule Nesfatin-1 [13] may be involved. Additionally, metabolic activation of neurons in preoptic areas of the hypothalamus has been found to be strongly related to homeostatic pressure for REM sleep in REM sleep deprivation studies [14]. Analysis of sleep patterns during REM rebound and recovery following sleep deprivation have suggested that REM sleep may be regulated on both short-term and long-term time scales [15], [16]. The longer time-scale regulates the daily amount of REM sleep and the short-term process dictates transitions between NREM and REM sleep during sleep episodes [15].

Taken together, the proposed mutually inhibitory network of neuronal populations governing transitions of REM sleep, coupled with a REM sleep homeostatic drive, mirrors the proposed flip-flop switch for sleep regulation composed of mutually inhibitory synaptic projections between the wake-promoting monoaminergic populations locus coeruleus (LC) and dorsal raphe (DR) and the sleep-promoting GABAergic ventral lateral preoptic nucleus (VLPO), under the influence of the putative adenosine-mediated homeostatic sleep drive [17]. How these two flip-flops may be coupled to produce transitions between wake, NREM and REM sleep observed in the majority of mammalian species is not completely determined. Synaptic projections from the wake-promoting LC and DR targeting REM-on and REM-off neuronal groups have been identified that act to suppress REM sleep [8], [18], [19]. However, limitations in recording simultaneously from these multiple areas at spatial and temporal resolutions to determine causal patterns of activity and interaction for transitions into and out of sleep and wake states hamper the ability to confirm or refute this and competing hypotheses of the sleep-wake regulatory network.

Recently, physiologically-based mathematical models have been introduced as a means to test the different and competing hypotheses for the sleep-wake regulatory network [20]–[25]. In previous work, we used a modeling formalism for the neurotransmitter-mediated interactions among wake-, sleep- and REM-sleep neuronal populations to investigate the cholinergic hypothesis for REM sleep generation in rats [24]. Here, we apply the same modeling formalism and develop a coupled flip-flop model to investigate how a wake/sleep flip-flop and a REM-on/REM-off flip-flop can interact to produce accurate behavioral state transition dynamics for the rat. We use experimental recordings of rat sleep behavior under ad libitum (baseline) conditions and during REM sleep rebound after 24 h of REM sleep deprivation to motivate a minimal set of projections between the flip-flops to account for sleep-wake patterns in both conditions.

The dynamics of a single mutually inhibitory flip-flop model have been well studied in the context of governing the transitions between wake and sleep states under control of a homeostatic sleep drive [21], [26], [27]. As we and others have shown previously, the dynamics are those of movement around a hysteresis loop with the homeostatic drive increasing and decreasing through bifurcation points to induce transitions between low and high population activity levels. As we discuss below, hysteresis loop dynamics have an inherent symmetry and regularity that may seem incompatible with the highly variable sleep-wake activity patterns of the rat. As in our previous modeling study of rat sleep-wake patterns [24], we include in our coupled flip-flop model several physiologically motivated stochastic components that introduce noise in our model solutions. To understand the implications of underlying hysteresis loop dynamics, we present a detailed analysis of the effects of the stochastic components on simulated bout durations for a single flip-flop model.

## Results

### Dynamics of a single flip-flop

In this section we briefly review the hysteresis loop dynamics of a single flip-flop and analyze the effects on these dynamics of physiologically motivated sources of noise. The goal of this analysis is to understand how the inherent symmetric and regular dynamics of a hysteresis loop can be modulated to simulate the variability of rat sleep-wake behavior, including the reported qualitative differences in the distributions of wake and sleep bout durations [28].

#### Hysteresis loop dynamics of a single flip-flop

**Figure 1:**
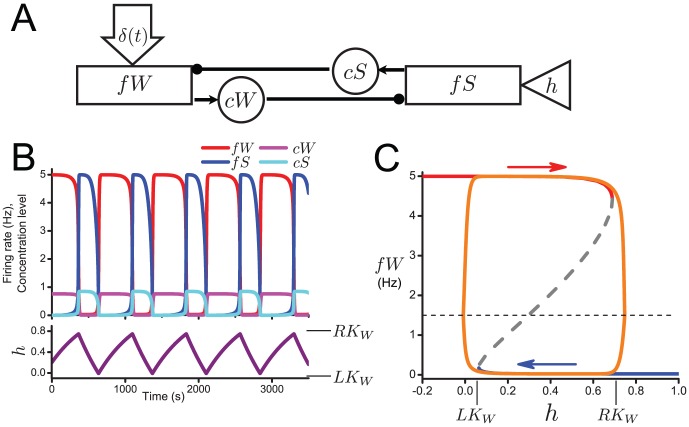
A: Schematic for the single wake/sleep flip-flop model. Rectangles represent neuronal groups and are labeled with their firing rate variables: 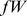 for wake-promoting and 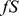 for sleep-promoting. Circles represent neurotransmitter concentrations expressed by the neuronal groups and are labeled with the variable names: 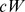 for the monoaminergic transmitters of the wake-promoting populations and 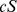 for GABA expressed by the sleep-promoting population. The triangle represents the homeostatic sleep drive (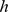). Solid lines ending in filled circles represent inhibitory synaptic projections. Large open arrow (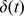) represents random excitatory stimuli to the wake-promoting population from sources external to the network. B: Time traces for 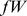, 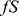, 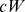, 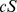 (upper panel) and 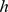 (lower panel). C: Bifurcation diagram of fixed point solutions of the fast subsystem (Eqs. (1), (2) with 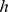 treated as a fixed parameter. The upper (red) and lower (blue) branches indicate stable solutions which are joined through a branch of unstable solutions (gray dashed) at saddle-node bifurcations occurring at 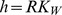 (right knee) and 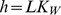 (left knee). The orange curve is the trajectory of the full model projected onto the 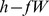 plane. Horizontal dashed line indicates 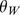, the 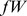 threshold defining the wake state.

We consider the wake/sleep flip-flop consisting of wake-promoting and sleep-promoting populations with reciprocal inhibitory neurotransmitter-mediated projections between them (Fig. 1A). The dynamics of the REM-on/REM-off flip-flop are analogous. In the figure, rectangles represent neuronal groups and are labeled with their firing rate variables, 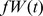 and 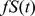, while circles represent neurotransmitter concentrations expressed by the neuronal groups and are labeled with their variable names, 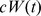 and 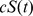. The wake/sleep flip-flop represents the mutually inhibitory synaptic interactions between the wake-promoting locus coeruleus (LC), dorsal raphe (DR) and the tuberomammilary nucleus (TMN) (jointly represented by 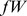) with the sleep-promoting ventrolateral preoptic nucleus (VLPO, 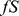) [17]. The model VLPO population expresses the inhibitory neurotransmitter GABA (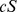) while the neurotransmitter expressed by the model wake population represents the joint effects of the transmitters expressed by LC, DR and TMN, namely norepinephrine, serotonin and histamine, respectively (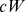). We constructed the model using our previously developed neuronal population firing rate and neurotransmitter formalism [24] (see Methods and Model section). Briefly, in this formalism, the firing rate of a pre-synaptic population, 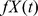 (in Hz), induces expression of neurotransmitter concentration, 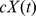, which, in turn, acts as input to post-synaptic populations (see Eqs. (1), (2) with 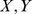  =  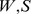).

In the flip-flop, transitions between sleep and wake states are governed by the homeostatic sleep drive, 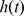, that describes the universally recognized propensity for sleep that increases during time awake and decreases during sleep, and is thought to involve the neuromodulator adenosine (reviewed in [29]). As such, 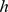 increases when 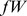 is at a high level (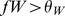) simulating the wake state (Fig. 1B, see Eq. (5)). Increasing values of 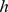 cause the sleep-promoting population to activate and force a transition to the state in which 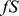 is at a high level and 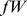 is at a low level, simulating sleep. As 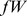 drops to a low level (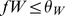), 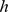 decreases until it deactivates 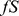 causing a transition back to the 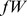 dominant state or simulated waking.

Relative to the time scale of transitions between the simulated waking and sleep states, the homeostat 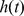 is slowly varying. For this analysis, we consider a separation of timescales, in which the variables 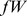, 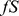, 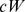 and 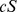 vary on a timescale faster than that of 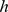. We call Eqs. (1) and (2) governing variables 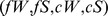 the fast subsystem, and Eq. (5) governing 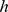 the slow subsystem. Here, we briefly summarize the intuition behind this fast-slow decomposition; for a rigorous review see [30]–[32] and for a previous analysis on a related model see [33].

We treat 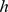 as a slowly varying parameter of the fast subsystem. For a fixed value of 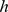, solutions to the fast subsystem approach a stable fixed point, the value of which may depend on the initial condition. The value of 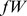 at this fixed point dictates if 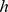 will increase or decrease in the full model. Slow variations in 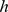 are instantly reflected by the convergence of the fast subsystem to a new stable fixed point. Thus, we may track the trajectory of the full model as a slow evolution through stable fixed point solutions of the fast subsystem.

The fixed point solutions, for 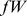, of the fast subsystem as a function of 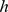 form a Z-shaped curve, as plotted in the bifurcation diagram in Fig. 1C. The upper (red) and lower (blue) branches are stable solutions. They are joined by an unstable branch of solutions (dashed) at saddle-node bifurcations occurring at a high value of 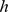, 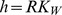 referred to as the “right knee”, and at a low value of 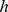, 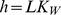 referred to as the “left knee”. Plotted on top of these fixed point solutions of the fast subsystem is the trajectory of the full model when 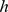 is allowed to evolve according to the slow subsystem (light blue curve). The Z-shaped curve of fast subsystem solutions forms the basis for hysteresis loop dynamics of the full model. When 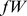 is at a high level (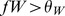, dotted line), 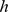 slowly increases and the fast subsystem variables remain attracted to the upper branch of fixed points. As 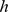 increases beyond the right knee of the curve at 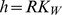, the only stable solution of the fast subsystem corresponds to low values of 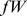 and the trajectory quickly approaches the lower branch of fixed points. On this branch 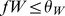, so 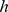 decreases and the full model trajectory tracks the lower branch of fixed points until the saddle-node bifurcation at 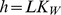. As 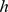 decreases below the left knee, the only stable solutions for the fast subsystem are on the upper branch and the trajectory jumps up, thus completing one cycle of the hysteresis loop, or one sleep-wake cycle.

#### Bout durations and the influence of variability

In the flip-flop model during one sleep-wake cycle, or one cycle around the hysteresis loop, wake and sleep bout durations are determined by the time the trajectory takes to traverse the upper and lower branches, respectively, of the Z-shaped curve of fixed point solutions of the fast subsystem (Fig. 1C). Thus, bout durations are governed by the distance 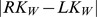 and the time dynamics of 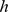. Asymmetry in wake and sleep bout durations can be introduced by different values of 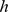 time constants during its increasing and decreasing phases, dictated by 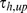 and 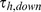 in our model. The time evolution of 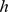, and thus bout durations, are further influenced by the values of 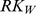 and 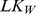 relative to the maximum and minimum limits on values of 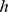, 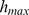 and 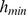, respectively. Since 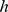 follows exponential dynamics, the evolution of 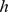 is slower when 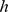 approaches either 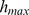 or 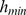, and is faster if 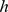 is near 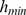 but increasing or 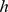 is near 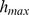 but decreasing. Thus, asymmetry in bout durations is also introduced due to the location of the Z-shaped curve within the interval 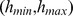.

As an example of how these two factors can compete in introducing asymmetry of bout durations, the longer wake bouts (460s) compared to sleep bouts (280s) shown in Fig. 1B were obtained with a faster 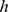 time constant during simulated wake (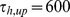
s) than during simulated sleep (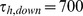
s), but with a shorter distance between 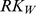 and 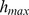 than the distance between 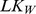 and 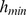. Hence, despite a faster time constant for 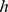 while the trajectory is on the upper branch of the Z-shaped fixed point curve, the evolution of 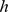 is slower than on the lower branch because of the proximity of 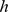 values to 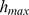.

A physiological flip-flop switch would be subject to different sources of variability. Here, we summarize our analysis of how physiologically motivated sources of noise, which we include in our models [24], perturb hysteresis loop dynamics; see Appendix S1 for complete details. First, we consider the effects of variability in neurotransmitter expression, modeled by multiplicatively scaling the steady state neurotransmitter expression functions 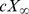 (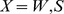) in Eq. (4) by the randomly varying term 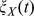. The amplitude of 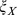 randomly varied (with uniform distribution and unit mean) at discrete times dictated by a Poisson process. In the single wake/sleep flip-flop model, 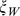 (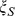) affects the steady state expression levels of monoamines (GABA) by the wake-promoting (sleep-promoting) population and thus modulates the level of synaptic inhibition between populations. As 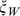, 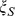 take on different values, in an interval around 1, they affect the Z-shaped fixed point curve, and thus the hysteresis loop, by changing the values of 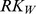 and 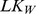. The distance between 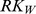 and 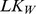 decreases for 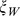 and 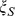 values less than 1 and increases for values greater than 1 (Fig. S1). This effect on the width of the hysteresis loop is a direct result of decreases and increases in inhibition between populations.

**Figure 2:**
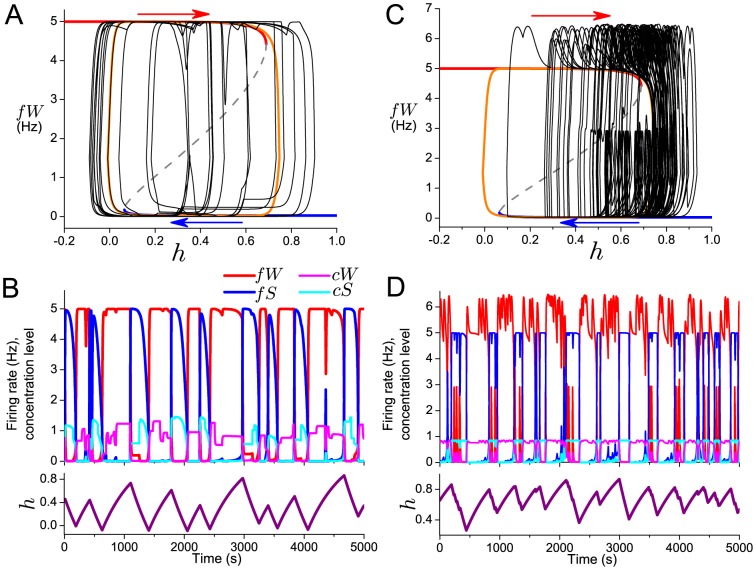
Effects of model variability on hysteresis loop dynamics of a single flip-flop. A, B: Variable neurotransmitter expression levels modulate position of the knees of the Z-shaped curve of fixed point solutions of the deterministic model (shown for reference with the deterministic trajectory (orange curve) in A). The projection of the noisy model trajectory (A: black curve) onto the bifurcation diagram illustrates the perturbations to the underlying hysteresis loop that result in variable wake and sleep bout durations illustrated in the time traces for 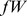, 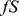, 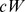, 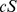 (B: upper panel) and 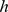 (B: lower panel). C,D: Random excitatory inputs to the wake-promoting population perturb the model trajectory (C: black curve) as it evolves around the deterministic hysteresis loop (Z-shaped curve of fixed point solutions and deterministic trajectory (orange curve) shown for reference in C), resulting in wake bouts with brief and longer durations, and sleep bouts with variable durations, as illustrated in the time traces for 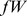, 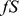, 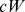, 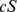 (D: upper panel) and 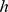 (D: lower panel).

As 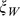 and 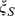 independently vary randomly around 1, the hysteresis loop randomly changes shape and position leading to variations in wake and sleep bout durations (Fig. 2A,B). Given that variations in 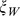 and 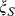 induce both lengthening and shortening of the hysteresis loop, one might expect that the variable bout durations would be symmetrically distributed about the bout durations dictated by the deterministic model. Our analysis indicates, however, that the distributions of bout durations depend sensitively on the relative time scales of the variability (i.e. the average frequency of 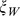 and 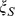 random variations) and the deterministic bout durations. In the noisy flip-flop model, the majority of bout durations are shorter than the durations of the deterministic model and they are distributed with a tail of longer durations (Fig. S2). Differences in the location of the Z-shaped fixed point curve in the 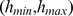 interval can introduce significant differences in the extent of the positive tail for wake or sleep bouts.

The second source of physiologically motivated variability we include in our model is brief, random excitatory stimuli input to the wake-promoting population, 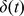, representing external inputs from brain areas not included in the model such as sensory or cortical areas. The inputs have random amplitude, decay with a fixed time constant and occur at discrete times dictated by a Poisson process. They do not modulate the hysteresis loop, but instead perturb the trajectory as it evolves around the deterministic hysteresis loop (Fig. 2C,D). If a stimulus occurs during a wake bout, the trajectory is perturbed to higher 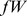 values which may extend the wake bout if the trajectory is close to 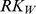. If the stimulus occurs during a sleep bout, it may result in a brief activation of 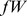 with a return to the sleep state, which we define as a brief wake bout, or result in a full transition to the wake state. Which result occurs is influenced by the position of the trajectory on the lower branch of the Z-shaped fixed point curve when the stimulus arrives. Stimuli occurring when the trajectory is closer to 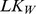 usually result in a transition to wake and stimuli occurring when the trajectory is closer to 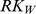 usually result in a brief wake bout. This dependence is due to whether the stimulus pushes 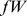 above the middle branch of unstable fixed point solutions which generally acts as a separatrix between the basins of attraction of the upper and lower branches of stable fixed point solutions.

The random excitatory stimuli to the wake-promoting population have different effects on the wake and sleep bout duration distributions (Fig. S2). The wake bout distribution is bimodal with a peak at very short durations indicating the brief wake bouts induced by the stimuli and a peak at longer durations for the bouts generated through hysteresis loop dynamics. The distribution of sleep bout durations takes on a more exponential-shape because sleep bouts are interrupted at random times by either brief wake bouts or early transitions to wake. Thus, an external input that preferentially targets one of the flip-flop populations can yield sleep-wake patterns with qualitative differences in wake and sleep bout durations.

### Coupled flip-flop model for rat sleep-wake regulation

As the above analysis suggests, a single flip-flop with multiple sources of variability and appropriately tuned parameters would be able to generate transition dynamics between states of wake and sleep, *or* between states of NREM and REM sleep, similar to those observed in the rat. The goal of this study was to investigate how a wake/sleep flip-flop and a REM-on/REM-off flip-flop can be coupled to reproduce transition dynamics among the three states of wake, NREM sleep and REM sleep in rat. To constrain model parameter settings and network structure, we analyze experimental recordings of rat sleep behavior under ad libitum (baseline) conditions and during REM sleep rebound after 24 h of REM sleep deprivation. Specifically, we consider data collected during a 4 h window in the light period (rest phase) at the same circadian phase for both conditions. We assume minimal modulation of sleep-wake behavior by the 24 h circadian rhythm during this 4 h window and, thus, do not include the influence of the circadian rhythm in the model. As we describe below, analysis of the data motivated a minimal set of projections between the flip-flops to account for sleep-wake patterns in both conditions. These projections include an effect of activity of the wake population on the REM sleep homeostat and an excitatory effect of activation of the REM-on population to the wake-promoting population.

**Figure 3:**
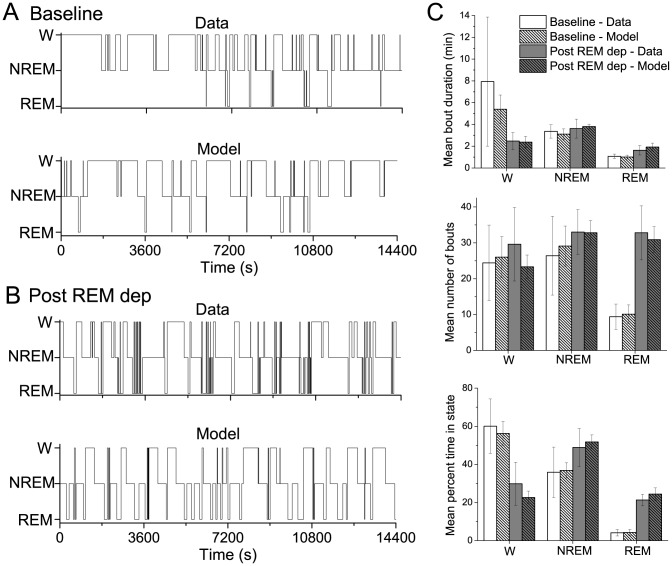
Hypnograms (A,B) and summary statistics (C) of experimental rat sleep recordings and model simulations in baseline sleep conditions and after 24 h of REM sleep deprivation. A,B: Representative experimental and simulated hypnograms depicting transitions between states of wake, NREM sleep and REM sleep in the baseline condition (A) and after REM sleep deprivation (B). C: Summary statistics, such as mean bout durations, mean number of bouts and mean percent time spent in state, for experimental data (unhatched bars) and model simulations (10 runs for 4 simulated h's, hatched bars) in the baseline (white bars) and post REM sleep deprivation (gray bars) conditions. No significant differences were computed between data and model results (paired, 2-tailed t-tests, 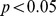).

As shown in Fig. 3, simulation results of our coupled flip-flop model were able to reproduce sleep-wake dynamics that were statistically similar to the experimentally recorded rat behavior under both conditions. Specifically, mean bout durations, number of bouts and percent time spent in each state for wake, NREM sleep and REM sleep, as well as probabilities (Table 1) were similar to the experimental data (paired, 2-tailed t-tests, 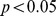). In this section, we first describe the key features of the experimental data that informed the model network structure and parameter settings. We then describe how the model was constructed to account for these key features, resulting in model dynamics similar to the data.

**Table 1:** Probabilities of behavioral state transitions.

		Data		Model	
		Baseline	Post REM-dep	Baseline	Post REM-dep
From Wake	To NREM	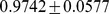	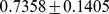	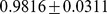	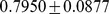
	To REM	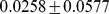	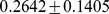	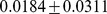	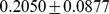
From NREM	To Wake	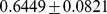	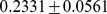	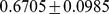	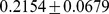
	To REM	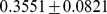	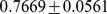	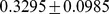	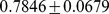
From REM	To Wake	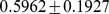	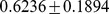	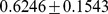	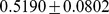
	To NREM	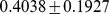	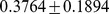	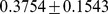	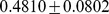

Probabilities of behavioral state transitions computed from the experimental sleep recordings under baseline and post-REM sleep deprivation conditions, and computed from 10 simulation runs of the coupled flip-flop model under each simulated condition.

#### Rat sleep-wake behavior in baseline conditions and after 24 h REM sleep deprivation

As previously reported for these experiments [34], rats showed a statistically significant increase in the percent time spent in REM sleep after 24 h of REM sleep deprivation compared to baseline conditions (Fig. 3C). Additional analysis revealed that the REM sleep increase was due to a large increase in the number of REM bouts as well as an increase in mean REM bout durations. The percent time spent awake also significantly decreased in the post-REM deprivation condition due to a trend towards shorter wake bouts. In particular, in baseline sleep conditions each rat had at least 1 wake bout of duration around 30 minutes, and 2 of the 5 rats had maximum wake bout durations of an hour or longer, accounting for the large variability in mean wake bout durations. In contrast, after REM sleep deprivation the longest wake bout any of the 5 rats exhibited was just over 30 minutes and the variability in durations was much reduced. This higher pressure for sleep in general and REM sleep particularly suggests that the REM sleep deprivation did not exclusively affect REM sleep homeostasis, but also influenced NREM sleep homeostasis.

Analyzing the probabilities of behavioral state transitions from the experimental recordings under baseline and post-REM sleep deprivation identified some key features of state transition dynamics that we used to construct the interactions between the wake/sleep and REM-on/REM-off flip-flops in our model (Table 1). As is characteristic of normal rodent sleep patterning, the probability was higher for the termination of a REM sleep bout by a transition into the wake state than by a transition to NREM sleep. The data suggests that the responsible mechanisms must be fairly robust as the probabilities were very similar in the two conditions. In the baseline condition, the data exhibited the normal sleep pattern in which sleep initiates in NREM sleep with the transition to REM sleep occurring after a latency period. Specifically, 4 out of 5 rats always transitioned from wake to NREM sleep. For the 1 rat that showed several wake-REM transitions, these transitions occurred exclusively as an interruption of REM sleep by a brief wake bout of duration 10–30 s, thus occurring as a REM-wake-REM transition. In the post-REM sleep deprivation condition, all 5 rats exhibited wake-REM transitions with the mean transition probability increasing to just over 25%. Again, these transitions occurred exclusively, in all rats, as REM-wake-REM transitions where the intervening wake bout was of brief duration (10–30 s). These findings suggest that the mechanisms by which REM sleep is terminated promote initiation of activity in wake-promoting populations but do not participate in the maintenance of that activity.

#### Replicating stereotypical wake-NREM-REM transition dynamics

**Figure 4:**
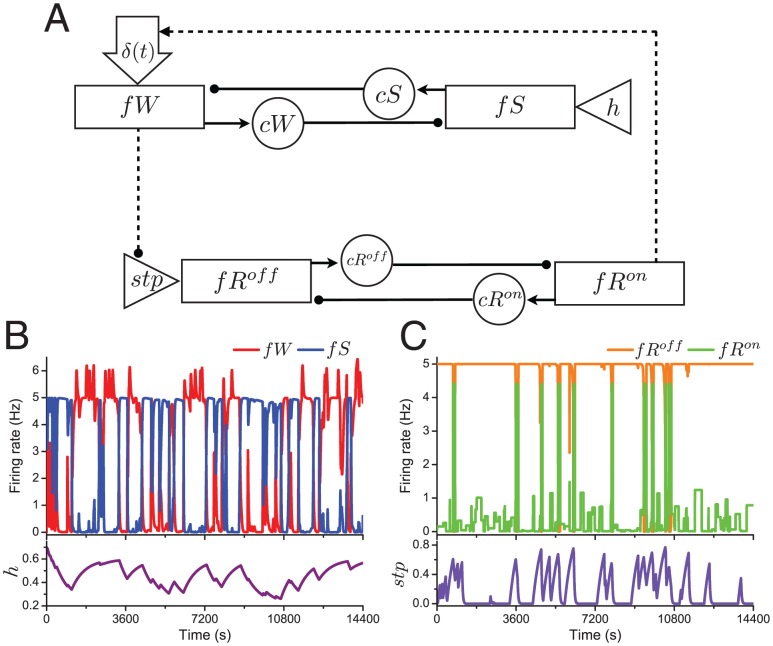
Schematic of the coupled flip-flop sleep-wake regulatory network model. Rectangles represent neuronal groups and are labeled with their firing rate variables: 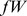 for wake-promoting, 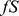 for sleep-promoting, 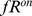 for REM-promoting (REM-on), 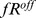 for REM-suppressing (REM-off). Circles represent neurotransmitter concentrations expressed by the neuronal groups and are labeled with the variable names: 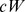 for the monoaminergic transmitters of the wake-promoting populations, 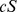, 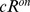 and 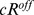 for GABA expressed by the sleep-promoting, REM-promoting and REM-suppressing groups, respectively. Triangles represent the homeostatic drives for sleep (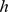) and REM sleep (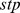). Solid lines ending in filled circles represent inhibitory synaptic projections; dashed lines indicate our proposed interactions between the flip-flops (filled circle indicates activity-suppressing, arrow indicates activity-promoting). Large open arrow (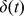) represents random excitatory stimuli to the wake-promoting population from sources external to the network. B,C: Time traces for 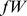, 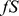 (B, upper panel), 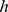 (B, lower panel), 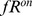, 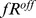 (C, upper panel) and 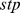 (C, lower panel) for the simulated baseline sleep shown in Fig. 3A.

In this section we describe how obtaining the stereotypical state transition pattern of wake to NREM sleep to REM sleep motivated the inclusion of an effect of activity of the wake population on the REM sleep homeostat in our coupled flip-flop model. As a starting point, we describe the dynamics of the coupled model using fast-slow decomposition. The coupled flip-flop model (Fig. 4A) consists of two pairs of neuronal populations, each pair reciprocally coupled by neurotransmitter-mediated inhibition, representing a wake/sleep flip-flop, with population firing rates and neurotransmitter levels modeled by Eqs. (1) and (2) with 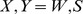, and a REM-on/REM-off flip-flop, with population firing rates and neurotransmitter levels modeled with Eqs. (1) and (2) with 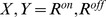. The REM-on population (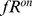) represents the joint activity of identified REM sleep-promoting populations and its neurotransmitter (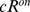) represents their joint GABAergic signaling. The REM-off population (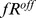) and its neurotransmitter (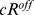) represent the GABAergic signalling of the identified areas with REM-off activity. Transitions in the sleep-wake flip-flop are governed by the homeostatic sleep drive, 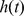, modeled by Eq. (5). As described above, 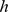 increases during waking (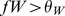) to promote activation of the sleep-promoting population and the transition into sleep, and decreases during sleep (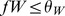) to promote deactivation of the sleep-promoting population. In the REM-on/REM-off flip-flop, transitions are governed by the REM sleep homeostatic drive modeled by the variable 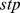, as a reference to the hypothesized short-term process involved in REM sleep homeostasis [15], using Eq. (6). To replicate the reported phenomena of REM sleep homeostasis, we model 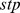 as increasing during NREM sleep (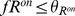) to promote deactivation of the REM-off population and the transition into REM sleep, and decreasing during REM sleep (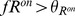) to promote activation of the REM-off population. This implementation of the REM sleep homeostatic drive is consistent with the concept that NREM-REM cycling is a sleep-dependent process [9], [15] and it generates cycling solutions that are robust to variations in parameter values, such as strength of inhibition between the REM-on and REM-off populations, as shown in previous analysis [27]. Additionally, we include variability in all neurotransmitter expression and brief random excitatory stimuli to the wake-promoting population (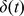 in Fig. 4A).

Qualitatively we can understand the dynamics of these two flip-flops during normal sleep behavior as follows (Fig. 4B,C): during the wake state, the wake-promoting population is activated and 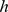 increases. To mimic the activity of identified physiological REM-off neuronal areas [8], the REM-off population is activated and the REM-on/REM-off flip-flop does not exhibit any transitions. When increasing 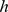 forces activation of the sleep-promoting population, a sleep episode is initiated, 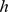 starts to decrease and 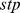 starts to increase. A sleep episode may be terminated at any time by deactivation of the sleep-promoting population as governed by 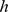 and a return to the wake state, or may be interrupted by a brief wake bout generated by the random excitatory stimuli to the wake-promoting population, 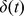. As a sleep episode continues, however, increasing 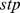 will deactivate the REM-off population, allowing the REM-on population to activate and a REM bout to occur. During a REM bout, 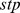 decreases and the REM bout can terminate due to activation of the REM-off population which returns the model to the NREM sleep state. Alternatively, the REM bout can be terminated by a brief wake bout. Eventually, the sleep episode ends when 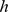 reaches a sufficiently low level resulting in deactivation of the sleep-promoting population and reactivation of the wake-promoting population.

To quantitatively understand transition dynamics in the coupled flip-flop model, we extend the fast-slow decomposition analysis to define two hysteresis loops that dictate trajectory dynamics, one defined by the wake/sleep flip-flop and the other by the REM-on/REM-off flip-flop. Informally, we consider both homeostatic drives as slow variables in the coupled flip-flop model with the homeostatic sleep drive 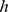 acting on a slower time scale than the REM sleep homeostatic drive 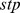. The firing rate and neurotransmitter level variables for the wake- and sleep-promoting, REM-on and REM-off populations (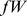, 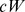, 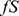, 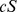, 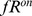, 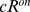, 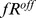, 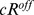) compose the fast subsystem. Since wake/sleep and REM-on/REM-off transitions occur during distinct phases of the trajectory and since each homeostatic drive only affects the fast variables of one of the flip-flops, we can separately apply fast-slow decomposition to each flip-flop to define two hysteresis loops. For the wake/sleep flip-flop, the fast-slow decomposition is identical to that described for the single flip-flop model, namely by considering 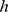 a fixed parameter in Eqs. (1) and (2) with 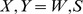, we obtain a Z-shaped curve of 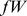 fixed point solutions. We refer to this curve as 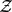. For the REM-on/REM-off flip-flop, we consider 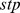 a fixed parameter in Eqs. (1) and (2) with 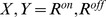 and obtain an S-shaped curve of fixed point solutions of 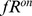, which we refer to as 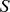. The reversal in the shape of the curve of fixed point solutions occurs because 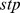 increases when 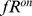 is deactivated (during NREM sleep). Solution trajectories of the model can be tracked in relation to these two fixed point curves, 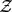 and 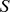, such that wake and sleep behavior corresponds to the trajectory evolving along the upper and lower branches, respectively, of 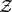 and, during sleep, REM and NREM episodes occur as the trajectory evolves along the upper and lower branches, respectively, of 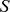.

To achieve activation of the REM-off population during wake and to robustly generate the stereotypical sleep pattern of wake-NREM-REM transitions that occurs after an extended period of waking, we need to include in the model a mechanism that forces the trajectory to remain on the lower branch of 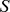 during wake and ensures that when the transition to sleep occurs, 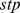 is at a value less than the right knee of 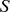. To determine these constraints on the model, we consider the physiological hypotheses for the action of the wake state on the REM sleep homeostatic drive. As discussed in [35], [36], there is debate whether the REM homeostatic drive increases during wake, decreases during wake or is unaffected by the wake state. In the context of our coupled flip-flop model, we can implement these hypotheses through the effect on 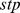 of activation of the wake population. If 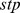 increases during wake, this may lead to an immediate (or almost immediate) transition from wake to REM, since 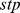 may reach values close to or above the right knee of 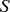 during an extended wake bout. Similarly, 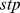 remaining constant during wake could also lead to immediate (or almost immediate) transitions from wake to REM. For example, as the data show, the majority of REM bouts end in a transition to wake. Thus, at the transition to wake 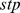 is at a level between the two knees of 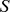, and could be at a value close to the right knee. Even if the trajectory is forced down to the lower branch of 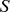 during wake in order to activate the REM-off population, when the next sleep episode occurs there could be a very short transition to REM as 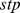 could quickly evolve to the right knee of 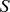. To guarantee a finite REM latency, as is stereotypical, we model that 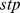 decreases during wake to a reset value 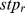. This mechanism corresponds to a saturating inhibitory effect of the wake state on the REM sleep homeostatic drive and insures that the REM-off population is activated during wake. We model this mechanism as an additional condition on 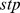 dynamics that when 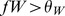 (wake), 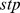 decays to 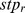 with a time constant 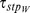, and when 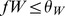 (sleep) 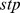 dynamics is given by Eq. 6 (see Eq. S15 in Appendix S1).

**Figure 5:**
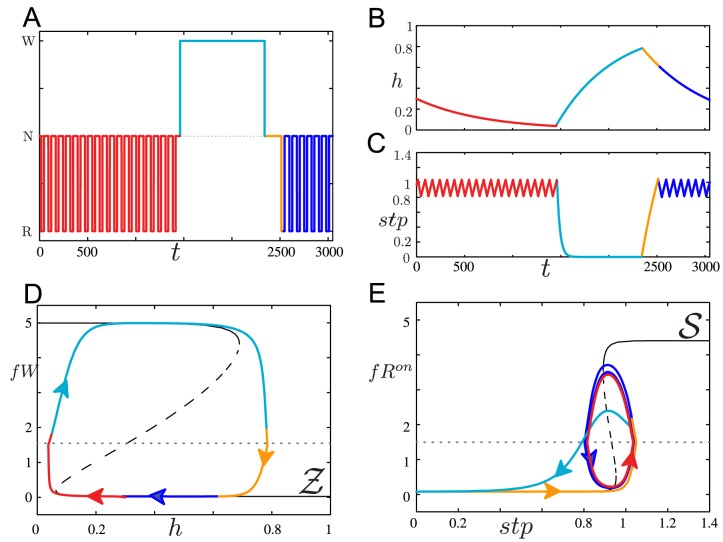
Coupled flip-flop model dynamics replicating wake-NREM-REM transition pattern. Hypnogram depicting state transition dynamics (A), homeostatic sleep drive 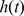 (B) and REM sleep homeostatic drive 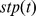 (C) in the deterministic model (all sources of variability removed) during REM/NREM cycling (red portion of curves) followed by a prolonged wake bout (light blue portion) and a subsequent return to REM/NREM cycling (orange and dark blue portion). D: Z-shaped curve of 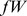 fixed point solutions (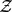) of the fast subsystem of the wake/sleep flip-flop with 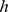 as a bifurcation parameter, with the projection of the trajectory of the full model onto the 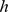-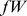 plane. The dashed line corresponds to the threshold 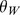 determining the wake state E: S-shaped curve of fixed point solutions (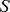) of the fast subsystem of the REM/NREM flip-flop, with 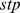 as a bifurcation parameter, with the projection of the trajectory of the full model onto 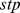-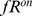 space.

To illustrate how this condition on 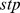 due to the wake state creates the stereotypical pattern of wake-NREM-REM transitions, we consider the trajectory of the deterministic model shown in Fig. 5 as a hypnogram (parameter values differ slightly than those in Table S1). At the beginning of the simulation, the model is in the sleep state exhibiting regular transitions between NREM and REM sleep (A, red portion of the curve). The trajectory is slowly evolving leftwards on the lower branch of 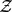 (D, red) as 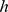 decreases (B, red), and 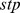 (C, red) is alternating between values associated with the left and right knees of 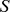 as the trajectory traverses the hysteresis loop defined by 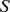 (E, red). When 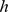 decreases below the left knee of 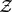, the wake/sleep flip-flop transitions to the wake state, interrupting the current REM or NREM bout (all panels, light blue). The trajectory jumps to the upper branch of 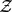, 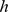 begins to increase and 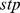 is driven down to 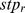 (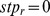 in this simulation), forcing the trajectory on the lower branch of 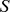. At the end of the wake bout as 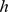 increases beyond the right knee of 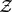, the trajectory jumps down to the lower branch of 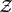, 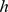 starts of decrease, 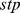 is released from its reset value 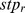 and begins to increase (all panels, orange). This portion of the trajectory represents the REM latency period. The first REM bout occurs when 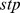 reaches the right knee of 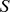 and the trajectory begins traversing the hysteresis loop defined by 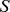 (all panels, dark blue). For values of 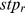 less than the left knee of 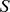, the REM latency period can be of longer duration than the interval between NREM and REM bouts determined by the hysteresis loop dynamics of 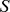.

In the full model with variability sources included, this inhibitory effect of the wake state on the REM sleep homeostatic drive remains a robust mechanism promoting the wake-NREM-REM sleep transition pattern. In particular, we set 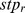 sufficiently low (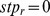) so that it remains less than the right knee of 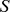 despite the modulation of the knees of 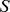 induced by the neurotransmitter variability.

There are other possible ways to robustly generate the Wake-NREM-REM transitions achieved here, such as including projections from the sleep-promoting population to the REM-on or REM-off populations with a decaying inhibitory effect or a decaying excitatory effect, respectively, that would prevent activation of the REM-on population at the onset of a sleep episode regardless of the influence of 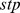. However, the inclusion of such additional projections would increase the complexity of the model and introduce new timescales and other parameters. In the absence of experimental hypotheses supporting such additional mechanisms, we focused on the effect of the waking state on the REM sleep homeostatic drive to generate the appropriate state transition dynamics.

#### Generating REM-wake transitions

Behavioral state transition probabilities computed from the experimental sleep recordings suggest a robust mechanism that terminates REM bouts with a transition to waking instead of NREM sleep. In the model, two mechanisms can generate transitions to the wake state from either the NREM or REM sleep state: either 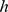 decays below the left knee of 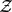 forcing the activation of 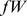 or an input to the wake-promoting population perturbs 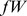 off the lower branch of 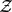 causing its activation and disruption of the sleep state. If these mechanisms are independent of the activity of the REM-on and REM-off populations, we expect that most sleep-wake transitions would occur from the NREM state because the model spends much more time in NREM compared to REM sleep. To generate REM-wake transitions, then, there could be an excitatory effect of activation of the REM-on population to the wake-promoting population, or an inhibitory effect to the sleep-promoting population. Within the context of our coupled flip-flop model, these excitatory and inhibitory effects could be direct synaptic projections from the REM-on population to the wake- or sleep-promoting population, REM-dependent actions on the homeostatic sleep drive or REM-dependent actions that indirectly influence transient or sustained external inputs to the wake- or sleep-promoting populations.

Investigation of these different effects in the model indicated that direct synaptic projections from the REM-on population to either the wake- or sleep-promoting population, or actions on the homeostatic sleep drive promoted the occurrence of wake transitions by modulating the hysteresis loop 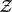 or 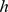 dynamics such that 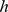 decreased below the left knee of 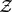, thereby causing the trajectory to jump to the upper branch of 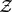. Such transitions from sleep to wake almost always led to an extended wake bout. Including a REM-dependent external input to either the wake- or sleep-promoting populations whose activity was sustained during REM bouts similarly affected model dynamics to cause transitions to extended wake bouts. While these mechanisms did not affect the ability of the model to replicate the experimental data in the baseline condition, it prevented replication of the post-REM sleep deprivation condition where REM-wake-REM transitions with brief intervening wake bouts were prevalent.

To investigate the effects of transient REM-dependent external inputs, we modulated the random excitatory stimuli to the wake-promoting population, 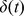, dependent on activation of the REM-on population. We found that model solutions fit the statistics of the experimental sleep-wake patterns in both baseline and post-REM sleep deprivation conditions when the frequency of 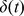 stimuli increased during the REM sleep state. Hence, the high REM-wake transition probability and the occurrence of REM-wake-REM transitions in the data indicated an indirect effect of REM-on activity on the wake/sleep flip-flop that initiated activation of the wake-promoting population but not maintenance of its activity. To implement this mechanism, we could include an additional source of transient excitatory stimuli to the wake promoting population that is only active during REM sleep. However, in the interest of keeping the model compact, we modeled the interaction through our existing mechanism 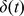. See the Discussion for possible physiological motivations for such stimuli. Alternatively, including a source of REM-dependent transient inhibitory inputs to the sleep-promoting population similarly resulted in high probabilities of REM-wake transitions and the occurrence of REM-wake-REM transitions but the mechanism was less robust as it depended on sufficient inactivation of the sleep-promoting population that could result in sufficient activation of the wake-promoting population.

We modeled the effect of REM-on activation on the random excitatory stimuli to the wake-promoting population, 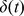, with an increase in the frequency of occurrence of the stimuli during REM sleep. In this way, although the model spends less time in REM sleep, more frequent stimuli to 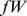 during REM induce more REM-wake transitions without introducing excessive brief wake bouts during NREM. When an excitatory stimulus to the wake-promoting population occurs during a REM bout, 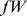 is perturbed off the lower branch of 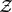. If 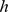 is sufficiently low, 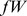 can transition to the upper branch of 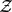, which forces 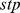 down to 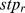 terminating REM-on activity and the wake bout will be maintained as 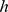 evolves along the upper branch of 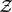. If 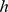 is closer to the right knee of 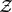 when the stimulus arrives during the REM bout, 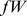 may be briefly perturbed off the lower branch of 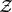 but it will return to the lower branch, thus generating only a brief wake bout. The sleep state that the model returns to depends on the rate of decrease of 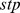 to 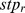 induced by the brief 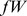 activation. If the rate of 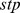 reset is fast, the model would return to the NREM sleep state because the trajectory would be forced below the left knee of 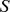 and 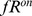 would deactivate during the brief wake. If the rate of 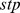 reset is slower, a REM-wake-REM transition can occur since 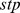 would have remained above the left knee of 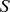 with perhaps only a transient reduction in REM-on activity.

#### Accounting for high variability of wake bout durations

In our experimental sleep recordings, especially under baseline conditions, some animals exhibited very long wake bouts. As described for the single flip-flop model, long wake bout durations can be obtained by adjusting time constants of 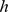 or providing the appropriate relationship between the saturation level of 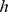 and the knees of the 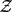. With variability of neurotransmitter expression included in the coupled flip-flop model, we can introduce very long wake bouts by setting the maximum saturation level of 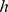, 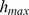, below the 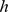 value of the right knee of 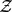. Without the neurotransmitter expression variability, these parameter settings would result in the model getting stuck in the wake state, because the model solution with 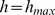 would be a stable fixed point of the full model. The neurotransmitter expression variability modulates the 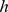 value of the right knee of 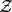 and can move it below 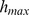 to induce a transition to the sleep state. Thus, in this parameter regime, wake bout durations are overall longer than sleep bouts, they have higher variability and very long wake bouts are possible. In the Discussion we provide possible physiological mechanisms that could support these model dynamics.

#### Differences between baseline and post-REM sleep deprivation sleep patterning

As shown in Fig. 3, the coupled flip-flop model is able to replicate sleep-wake patterning under both baseline and post-REM sleep deprivation conditions. The significant differences in patterning between the two conditions include increases in percent time spent in REM sleep, the number and duration of REM sleep bouts and a decrease in the percent time spent in waking (2-tailed paired t-test, 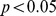). We captured these differences in the model by adjusting parameters governing three model components. First, we increased the number and duration of REM bouts by adjusting the maximum and minimum saturation levels of 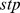. The maximum saturation level, 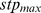, was increased to promote shorter latencies to REM-on activation and the minimum level, 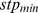, was increased to slow down the trajectory's evolution on the upper branch of 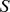 during a REM bout. To further affect REM bout durations, we lengthened the REM-NREM hysteresis loop by increasing the distance between the knees of 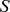. In our model formalism, the distance between the knees of 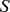 can be modified in different ways, including the addition of external inputs to either the REM-on or REM-off populations. Alternatively, we adjusted the influence of 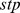 on 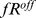 activity (see Appendix S1 for details). An additional consequence of a longer REM-NREM hysteresis loop is an increase in REM-wake-REM transitions, which also was a feature of post-REM sleep deprivation sleep patterning. As described above, REM-wake-REM transitions occur in the model when a random excitatory stimulus arrives to the wake-promoting population during a REM bout. As a result of brief 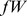 activation, 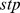 is forced to decrease towards the reset value 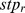. However, when the knees of 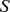 are further apart, the decrease in 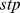 is less likely to push 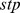 below the left knee of 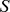 during the brief wake bout. When the brief wake bout ends, the REM-on/REM-off flip-flop remains in the REM state and REM bouts are more robust to this kind of interruption.

Secondly, we obtained the decrease in percent time spent in waking as a result of REM sleep deprivation through a decrease in wake bout durations. Specifically, we increased the maximum saturation level of 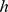, 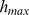, to a value above the right knee of 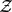. As described above, this shortened wake bouts by allowing 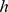 to freely evolve past the right knee of 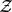 to induce the deactivation of 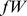 and end the wake bout. The third model component we adjusted to capture post-REM sleep deprivation patterning was to reduce the frequency of the random excitatory stimuli to the wake-promoting population. A large reduction in stimulus frequency during the wake and NREM states resulted in far fewer brief wake bouts during NREM sleep that fragmented NREM and prevented the increase of the REM sleep homeostatic drive 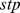. Therefore, we obtained the large increase in the NREM to REM transition probability exhibited in the sleep recordings (Table 1). The reduction in stimulus frequency during REM sleep, while not as great, reduced the interruption of REM bouts by terminating stimuli. In the Discussion, we provide possible neural mechanisms that could account for these parameter changes.

## Discussion

The proposed conceptual models for the control of REM sleep by mutually inhibitory networks of REM-on and REM-off populations leave a number of questions unanswered, particularly regarding the interactions between the populations controlling REM sleep and those controlling wake and NREM sleep [5], [8], [19]. In an attempt to shed some light on these questions, we constructed a simplified, yet cohesive, mathematical model based on mutually inhibitory flip-flop networks for the control of state transitions between wake, NREM and REM sleep states. We utilized a modeling formalism that correlates with the physiological network structure of neurotransmitter-mediated interactions among state-promoting neuronal populations. The network structure, in particular the interactions between the wake/sleep flip-flop and the REM-on/REM-off flip-flop, was motivated by the behavioral state transitions observed in experimental rat sleep recordings. A minimal set of interactions was identified by constraining the network model to accurately replicate experimental state transition dynamics in both baseline sleep and post-REM sleep deprivation recovery sleep in a consistent manner. As discussed below, these modeled interactions predict physiological mechanisms that can be targeted in future experimental studies to more definitively address some of the unanswered questions of REM sleep regulation.

A strength of this study was the use of experimental rat sleep recordings to motivate the construction of the network model. We relied on replication of summary bout statistics and probabilities of behavioral state transitions to constrain the model. Recently, higher order statistical approaches, such as survival-based analysis of wake and sleep bout durations, have been applied to sleep recordings to identify effects of disease states and experimental manipulations [37]–[40]. To compare survival analyses of bout durations of the data and model results requires a sufficient number of bouts [41]. Since our data sets contained only five 4 h recordings in each condition, baseline and post-REM sleep deprivation, there were insufficient numbers of bouts, particularly REM sleep bouts, for a bout duration survival analysis to be meaningful. We note, though, that the standard summary statistics and state transition probabilities were sufficient to rule out possible interactions between the wake/sleep and REM-on/REM-off flip-flops. Specifically, to robustly generate transitions from REM sleep to wake, an alternative interaction between flip-flops is a direct excitatory projection from the REM-on population to the wake population. However, simulations with this alternative model structure were not able to replicate all summary statistics and behavioral state transition probabilities. In particular, this alternative model could not simultaneously replicate the correct REM-wake transition probability and number of REM bouts in either the baseline or post-REM sleep deprivation condition. In this alternative model, REM-wake transitions most often resulted in an extended wake bout instead of a brief wake bout as part of a REM-wake-REM transition that occurred more often in the data. Thus, we believe that summary statistics and transition probabilities were adequate to constrain our simplified model.

Mathematically, a mutually inhibitory flip-flop network possesses the dynamics of a hysteresis loop. The inherent symmetry of a hysteresis loop and the regularity of its dynamics may call into question its suitability for replicating the highly variable state transitions of rat sleep-wake behavior. For example, survival analysis of wake and sleep (NREM and REM sleep combined) bout durations of rodent sleep revealed an asymmetry between wake and sleep bouts such that wake bout durations displayed power-law like distributions while sleep bout durations exhibited exponential distributions [28]. Such a qualitative difference in bout duration distributions suggests that wake and sleep state transitions are influenced by different mechanisms. Our analysis of the dynamics of a single flip-flop network with physiologically motivated sources of variability included suggests that it can generate sufficient variability and asymmetry in wake and sleep bout duration distributions. In particular, an extended tail in wake bout distributions reflecting low numbers of very long wake bouts could be introduced by the appropriate relationship between the underlying hysteresis loop and the saturation level of the homeostatic sleep drive. Additionally, brief excitatory inputs to the wake population generated a bimodal wake bout distribution and an exponential-like sleep bout distribution. Recent work has suggested that wake distributions may follow a multi-exponential distribution rather than a strict power-law distribution [42]. One can imagine that in the proper parameter regime, a bimodal wake bout distribution with an appropriate tail, as we have shown a single flip-flop with noise sources can generate, may result in a multi-exponential-like distribution. Further work fitting flip-flop models to survival analysis of experimental sleep data is clearly needed to assess the capability of this network structure to account for all aspects of sleep state transition dynamics.

### Comparison to previous flip-flop models of the sleep-wake regulatory network

Previous modeling studies have investigated a coupled flip-flop network for sleep-wake regulation in humans [23], [25]. Our study differs in that we focus on accounting for the variable and polyphasic sleep-wake activity typical of rats rather than a caricature of stereotypical human sleep-wake activity. A more important difference involves the modeling formalism. The previous studies modeled the mean activity or firing rate of each neuronal population with the Morris-Lecar model, a generic model for single cell neuronal membrane potential [31], [43]. When parameters are tuned appropriately, the Morris-Lecar model can generate fast transitions between states of low and high activity that is appropriate for modeling the activation and deactivation transitions of mean activity in neuronal populations. As a model for neuronal action potential generation, the Morris-Lecar model has the additional capability of generating oscillatory solutions. In the translation of using the model for population activity, this means that the model can generate spontaneous, regular transitions between low and high activity levels, without any external stimulation. Both the Rempe et al [23] and Kumar et al [25] studies exploit the model's capability of intrinsic population oscillatory dynamics to account for NREM-REM transitions. In the modeling formalism that we use, on the other hand, individual cell groups do not have the capability of intrinsic oscillatory behavior. Transitions between low and high activity levels depend on changes in external inputs, specifically homeostatic drives for NREM or REM sleep. Thus, in our model results, NREM-REM transitions occur in response to changes in explicitly defined model components, instead of assumed population properties. As the aim of our study is to propose potential physiological interactions among behavioral state-promoting neuronal groups, we believe that it is important to avoid incorporating extraneous assumptions into our modeling formalism.

In these previous models, the coupling projections between the wake/sleep and REM-on/REM-off flip-flops were similarly motivated by generating appropriate state transition dynamics, particularly the stereotypical transition pattern of wake to NREM sleep to REM sleep. The Kumar et al [25] model primarily achieves this pattern by the action of a REM sleep homeostatic drive whose dynamics were sensitive to wake and sleep states. This drive increased excitation to the REM-on population during sleep to promote its activation and decreased excitation during wake, which allowed the REM-off population to dominate at wake to sleep transitions. The Rempe et al [23] model included an indirect projection from the wake/sleep to the REM-on/REM-off flip-flop through an intermediary population, the extended VLPO (eVLPO). Wake and sleep dependent activity of the eVLPO gated activity of the REM-off population during wake and sleep states in order to promote its initial activation at the wake to sleep transition. Both models include additional feedforward connections between flip-flops, such as direct inhibitory projections from the wake-promoting population to the REM-on population, that are suggested by anatomical studies and work to suppress REM-on activity during wake. We note that inclusion of similar additional feedforward projections from the wake/sleep flip-flop to the REM-on/REM-off flip-flop in our model would have similar effects and not qualitatively change our results. In both the Rempe et al and Kumar et al models, replicating human sleep-wake patterns did not constrain the form of feedback projections from the REM-on/REM-off flip-flop to the wake/sleep flip-flop. While an indirect feedback projection originating in the REM-off population was included in the Kumar et al model, it was not necessary to obtain appropriate model behavior, and the Rempe et al model did not include any feedback projections.

Since the Rempe et al [23] and Kumar et al [25] models simulated human sleep, they both included input from a circadian oscillator to contribute to the 24 h modulation of sleep-wake behavior. In this study, we focused on replicating variable and polyphasic rat sleep-wake behavior during a 4 h window in the rest phase, assuming minimal modulation by the circadian rhythm. Our modeling formalism, however, can readily include a neuronal population representing the suprachiasmatic nucleus (SCN) whose activity is driven by a circadian oscillator and which is coupled to the sleep- and wake-promoting populations within the network. Indeed, our previous work modeling rat sleep-wake behavior, in which REM sleep is generated by a reciprocal interaction network, suggested that multiple signaling pathways between the SCN and sleep-wake centers may be necessary to account for circadian modulation of rat sleep [44]. As a direction for future work, incorporation of the SCN circadian signal in the coupled flip-flop model to account for the 24 h variation of rat sleep-wake behavior would test the minimal interactions between the wake/sleep and REM-on/REM-off flip-flops proposed here and perhaps identify additional constraints on the network structure.

### Model predictions

To obtain robust wake-NREM-REM transition dynamics, our simplified coupled flip-flop network model predicts that during waking, the REM sleep homeostatic drive resets to a level corresponding to low REM sleep pressure. This prediction is similar to that of Benington and Heller [35] that REM sleep need is homeostatically related to NREM sleep rather than waking, such that in normal conditions it accrues during NREM and not waking. While Benington and Heller do not offer a mechanism defining the behavior of the REM sleep homeostat during the transition from sleep to wake, we found that providing a decay of the REM sleep homeostat to the reset level during wake best replicated rat sleep patterning. In the context of the coupled flip-flop model, this mechanism most parsimoniously provides the appropriate dynamics to ensure that after an extended wake bout, the NREM sleep state is entered first before REM sleep occurs. A physiological correlate of such a mechanism could be provided by the expression during sleep states of a substance mediating REM sleep need and its cessation during waking. The absence of expression during wake would lead to the degradation or uptake of the substance, decreasing its presence to low levels. Recent experimental results indicate that expression of MCH and Nesfatin-1, which are co-expressed by neurons in the tuberal hypothalamic area, exhibit sleep-dependent increases and wake-dependent decreases and have REM sleep-inducing effects [12], [13], [45]. Interestingly, while these substances are co-expressed, pharmacological experiments indicate that MCH promotes REM sleep [45] while Nesfatin-1 suppresses REM sleep [13]; however, recent optogenetic experiments report that acute activation of MCH neurons promotes maintenance of REM sleep [46]. An alternative physiological mechanism could be provided by sleep-dependent increases in activity of neurons that promote a transition into REM sleep. Neural activity in the median preoptic nucleus (MnPN) and the ventral lateral preoptic area (vlPOA) strongly correlates with REM sleep pressure [14]. These neural groups show higher activity during sleep than during waking which could mediate a wake-dependent decrease in REM sleep homeostatic drive. It is most likely that the physiological mechanism for a REM sleep homeostatic drive is more complicated than the simple, single drive variable included in the model. For example, experiments in cats have suggested that REM sleep pressure during REM deprivation and REM sleep rebound during the recovery from REM deprivation may be governed by mechanisms in separate brain areas [47]. Further experimental investigation can provide insight for a more accurate model.

The robust propensity for REM bouts to terminate in a transition to wake exhibited in the data suggests the existence of an effect on wake-promoting populations by the activation of REM-on populations. However, while the anatomy has not been completely determined, there is little evidence of direct synaptic projections from the key identified REM-on populations to wake-promoting areas. For example, the efferent pathways of the SLD descend to areas that govern motoneuron activity to control muscle atonia during REM, and ascend to thalamic areas that induce REM cortical activation (reviewed in [8]). Thus, an indirect mechanism whereby wake-promoting populations are activated to terminate REM may be likely. We arrived at this conclusion by constraining the model to replicate transition dynamics of the data. As described above, model dynamics accurately replicated all summary statistics and probabilities of behavioral state transitions when activation of the REM-on population increased the external excitatory stimuli to the wake-promoting population, but not when it had a direct excitatory effect on the wake-promoting population. A physiological correlate for the model mechanism could be provided by top-down projections to wake-promoting populations from cortical regions that are activated during REM sleep, but not NREM sleep. An alternative interpretation is that the wake-promoting population is more sensitive to external inputs during the REM sleep state, leading to a higher probability that wake-promoting populations will be briefly activated during REM sleep. Dynamically, increasing the rate of external excitatory stimuli during REM sleep is essentially equivalent.

In the model, the occurrence of very long wake bouts and high variability of wake bout durations were achieved by allowing the homeostatic sleep drive 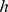 to reach its saturating limit 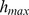 and relying on neurotransmitter expression variability to modulate the hysteresis loop to induce the transition out of the wake state. While this may not be a physiologically robust mechanism, it is, however, dynamically similar to the presence of a wake-promoting factor, such as orexin (see [48] for a review), that could disrupt or delay homeostatically governed transitions to sleep. The time-dependence or variability in the expression of such a wake-promoting factor would then determine the transition out of sleep and thus the duration of the wake bout in a manner similar to random variations of neurotransmitter levels included in the model.

The effects of 24 h REM sleep deprivation on rat sleep behavior were replicated in the model by modulating both the sleep and REM sleep homeostatic drives. Dynamics of the sleep homeostatic drive were modulated to promote transitions into sleep from waking. The need for this modulation may reflect a direct effect on sleep homeostasis by the REM deprivation protocol due to disturbances or slight losses of total sleep. For the effect on the REM sleep homeostatic drive, REM bouts were lengthened and, consequently, resistance to REM interruption was strengthened by modulating the influence of the REM homeostat on activation of the REM-off population. Such modulation could be a mechanism for the proposed long-term process that regulates the daily amount of REM sleep [15]. Physiologically, it might reflect modulation of receptors for substances mediating short term REM homeostasis, such as MCH and Nesfatin-1, as a result of REM deprivation. This type of modulation may also be a mechanism to account for the resistance to REM interruption achieved by the reduction in the frequency of excitatory stimuli to the wake-promoting population that was implemented to account for the effects of REM sleep deprivation.

### Conclusions

In this study, we proposed a minimal model of an inhibition based network for the regulation of transition dynamics between the states of wake, NREM and REM sleep. We readily concede that the model may be too simple. Our intent, however, was to provide a cohesive inhibitory network structure, based on known physiology, and constrain the structure to account for experimentally measured sleep patterning in baseline conditions and after an experimental challenge, namely REM sleep deprivation. Key model results are predictions of the interactions between the subnetworks controlling sleep-wake transitions and REM-NREM transitions: feedforward effects from the wake/sleep subnetwork to the REM/NREM subnetwork that act to suppress REM propensity during waking, and feedback effects that indirectly promote the initiation, but not maintenance, of waking as a result of REM sleep. The aim of this modeling study, similar to other recent physiologically-based modeling work on sleep-wake regulatory networks [20]–[25], is to participate in the investigation of neuronal regulation of sleep and play the same role of providing a framework for understanding and interpreting experimental observations as the phenomenological two-process model [49] and the reciprocal interaction model [3] have done for the past 40 years, but in the context of our increased knowledge of the underlying physiology of sleep-wake regulation.

## Methods and Model

### Experimental sleep recordings and sleep scoring

#### Ethics statement

Experimental procedures were approved by the University of Michigan Committee for the Care and Use of Animals (permit #08194) and were in accordance with the National Institutes of Health Guide for the Care and Use of Laboratory Animals. All surgery was performed aseptically under sodium pentobarbital anesthesia, and every effort was made to minimize suffering.

Recordings of rat sleep under baseline conditions and after 24 h of REM sleep deprivation were conducted as previously described [34]. Briefly, six male Fischer 344 rats (Simonsen Laboratories, Gilroy, CA) were used in the study. Under sodium pentobarbital anesthesia, the following electrodes were implanted by aseptic surgery for the purpose of electroencephalography (EEG) recording and analysis of behavioral state: 2 superficial cortical electrodes (relative to bregma AP: +0.3 and ML: +1.0 for left frontal; AP: -3.0 and ML: −2.0 for right parietal), 1 deep electrode targeted to the left dorsal hippocampus (AP: −3.0, ML: +2.0 and DV: −2.9), 1 sinus ground and 1 recording wire into each dorsal neck muscle to record nuchal electromyography (EMG). After recovery from surgery and habituation to the recording apparatus, each rat was recorded under the following conditions: (1) 8 h of natural sleep-wake behavior, and (2) 8 h of natural sleep-wake behavior, preceded by 24 h of REM sleep deprivation. Animals were deprived of REM sleep using the multiple platforms-over-water method [50]. Recordings commenced 2 hours into the light phase of the 12-hour light:12-hour dark cycle (lights on at 6:00am) to which the animals were habituated.

EEG and EMG recordings were analyzed to determine behavioral state. Each 8 h recording session was analyzed in 10 s epochs. Three trained experimenters were blinded to the condition of each rat and scored each record for REM sleep, NREM sleep and waking [51]. The average pair-wise agreement for the three scorers was 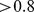 for total sleep, NREM sleep and REM sleep. REM sleep was identified by low amplitude and desynchronized cortical EEG, synchronized hippocampal EEG in the 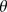 band (4–9 Hz) and quiescent EMG. Sleep-scored data from 5 animals were used in this study as the sleep patterning statistics (such as mean bout duration, number of bouts and percent time spent in each state) of the 6th animal differed by more than 2 standard deviations from the average sleep patterning statistics of the other 5 animals.

To avoid effects due to recording initiation and circadian modulation, sleep-scored data for hours 2–5 of the 8 h recording for each rat (n = 5) were used for model development.

### Model formalism

We constructed the single flip-flop and coupled flip-flop models using our previously developed neuronal population firing rate and neurotransmitter formalism [24]. In this formalism, the firing rate of a pre-synaptic population, 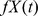 (in Hz), induces expression of neurotransmitter concentration, 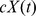, which, in turn, acts as input to post-synaptic populations. For a single flip-flop pair of populations, the firing rate and neurotransmitter equations are
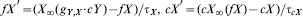
(1)

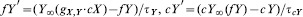
(2)where 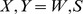 for the wake/sleep flip-flop or 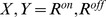 for the REM-on/REM-off flip-flop. Since we consider only inhibitory influences of all neurotransmitters, the weighting parameters for the post-synaptic influence of all neurotransmitter 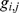 are negative. The time constants 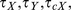
and 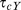 reflect the time dynamics associated with firing rate response and neurotransmitter expression, respectively, at the population level. Because different experimental techniques lead to differences in absolute reported neurotransmitter concentrations, we normalize each neurotransmitter concentration between 0 and 1. The functions 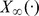 represent the steady state activation functions for population firing rates and are given by the standard sigmoidal form of neuronal firing rate models [52], [53]

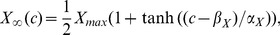
(3)where 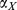 and 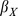 govern the slope and half-activation level of the activation function, respectively. The functions 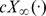 represent the steady state expression functions for neurotransmitter concentrations and follow a saturating profile 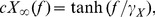
(4)with slope dictated by 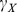. In simulations of the model networks, states of wake, NREM sleep, and REM sleep were interpreted based on firing rates of neuronal populations and concentration levels of their associated neurotransmitters.

In each flip-flop, transitions between states are governed by a homeostatic drive that promotes sleep in the wake/sleep flip-flop and promotes REM sleep in the REM-on/REM-off flip-flop. The sleep-promoting homeostatic drive, 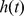, describes the universally recognized propensity for sleep that increases during time awake and decreases during sleep, and is thought to involve the neuromodulator adenosine (reviewed in [29]). The REM sleep-promoting homeostatic drive, 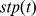, represents the proposed short-term process underlying REM sleep homeostasis [15], and while its underlying physiological mechanisms have not been identified, we model it similarly as the sleep-promoting homeostat such that 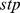 increases during NREM sleep and decreases during REM sleep. The equations governing the homeostatic drives are: 
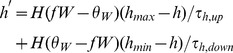
(5)

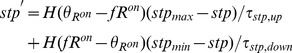
(6)where 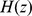 is the Heaviside function defined as 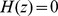 if 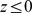 and 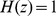 if 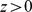, and 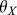 (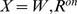) are 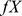 threshold values indicating the occurrence of wake or REM sleep, respectively. The parameters 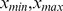 (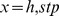) give the minimum and maximum values, respectively, that the drive variables can attain and 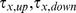 dictate the time scales of their increase and decrease, respectively. To incorporate the sleep-promoting homeostatic drive into the wake/sleep flip-flops, we model the effects of adenosine on the VLPO [54]–[56] by setting the half-activation level of the sleep population's steady-state activation function, 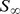, to be dependent on 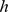. Since there is less physiological evidence to support how the REM-sleep homeostatic drive affects either the REM-on or REM-off populations, we turn to a previous analysis of flip-flop models for REM sleep control that indicated that transition dynamics are more robust when the REM sleep-promoting homeostatic drive acts on the REM-off population [27]. Thus, we set the half-activation level of the REM-off steady state activation function, 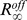, to be a function of 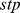. The equations for the sleep and REM-off steady state activation functions are as follows (compare to Equation (3)): 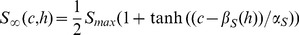
(7)

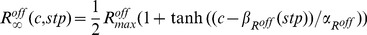
(8)where 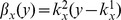 for 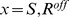 and 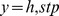, respectively. For the wake/sleep flip-flop, 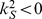 such that as 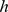 increases during wake, 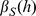 decreases to promote activation of the sleep population and terminate the wake bout. For the REM-on/REM-off flip-flop, 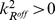 such that as 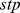 increases during NREM sleep, 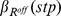 also increases to promote inactivation of the REM-off population and allow the REM-on population to activate.

A full listing of model equations and parameter values is given in Appendix S1 and Table S1.

### Sources of variability in the model

To incorporate experimentally-documented variability of neurotransmitter release into the model [57], we multiplicatively scaled the steady state release functions for each neurotransmitter, 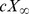, by a noise factor, 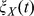 whose amplitude randomly varied (with uniform distribution and unit mean) at discrete times dictated by a Poisson process. Another source of variability included in the model represents external excitatory stimuli to the wake population resulting in brief wake bouts that fragment sleep states as is characteristic of rat sleep patterning. These inputs are modeled by the addition of random amplitude, brief excitatory inputs occurring at discrete times dictated by a Poisson process, 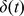, in the argument of the steady-state activation function of the wake population, 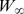.

### Interactions between the wake/sleep and REM-on/REM-off flip-flops

As described above, analysis of the dynamics of state transitions in the experimental sleep recordings under baseline conditions and during recovery sleep following REM sleep deprivation informed the interactions between the wake/sleep and REM-on/REM-off flip-flops included in the model (Fig. 4A). Activity of the wake population influences the REM sleep homeostatic drive 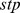 such that when the model is in the wake state, 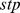 decays to a low level, 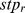, forcing the half-activation of the REM-off population, 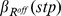, to a low level and promoting its activation. When the wake population is inactive, 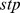 dynamics are governed by Eq. (6) (see Eq. S15 in Appendix S1). We model an indirect effect of activity in the REM-on population on the wake population such that when the model is in REM sleep, the frequency of the external excitatory stimuli targeting the wake population, 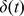, is increased.

### Numerics

Statistical analysis was performed in the MATLAB software package (The MathWorks, Natick, MA). The administrative code was also written in MATLAB. Differential equation integration was performed using the XPP software package [58] (www.math.pitt.edu/bard/xpp/xpp.html), interfaced with MATLAB via system calls. Two dimensional bifurcation diagrams were created using XPP, and the higher dimensional diagram in Fig. S1B was created using MATCONT (www.matcont.ugent.be).

## Supporting Information

Figure S1
**Modulation of the single wake/sleep flip-flop model by neurotransmitter expression variability.** Modulation of the single wake/sleep flip-flop model by neurotransmitter expression variability. A: 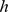 coordinates of the right (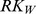) and left (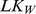) knees of the hysteresis loop as 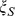 is varied (black dotted curve, 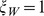) and as 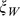 is varied (red solid curve, 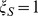). B: Distance between 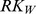 and 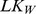 as both 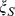 and 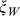 are varied. Solid colored bands are contours indicating equal distance between the knees. White x's indicate values where the knees do not exist.(EPS)

Figure S2 **Effects of model variability on distributions of wake and sleep bout durations.** Effects of model variability on distributions of wake (A,C) and sleep (B,D) bout durations. A,B: Variable neurotransmitter expression levels result in positively skewed, Gaussian-like distributions with a positive tail of long wake bouts. C,D: Random excitatory inputs to the wake-promoting population result in a bi-modal distribution of wake bout durations (C) and an exponential-like distribution of sleep bout durations (D). Bout durations of the deterministic model are indicated by vertical black lines in all plots; distributions were normalized by the area under their curves.
(EPS)

Figure S3
**Modulation of the REM-NREM hysteresis loop in the post-REM sleep deprivation condition.** Values of 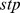 at the right knee (upper curve, 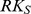) and at the left knee (lower curve, 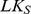) of the S-shaped curve of fixed point solutions (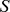) of the fast subsystem of the REM/NREM flip-flop, as a function of 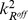. Observe that the 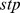 interval between the knees (vertical distance between curves) grows as 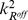 decreases from the baseline value of 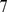.(EPS)

Appendix S1 **Full model equations, analysis of the effects of model variability on the dynamics of a single flip-flop model, and analysis of the differences between the model baseline and post-REM sleep deprivation conditions.**
(PDF)

Table S1 **Model parameter values for baseline sleep case.**
(PDF)
