# Supplementary material for: Coupled Flip-Flop Model for REM Sleep Regulation in the Rat
Source: PLoS One. 2014 Apr 10;9(4):e94481. doi: 10.1371/journal.pone.0094481 (PMC3983214; doi:10.1371/journal.pone.0094481)
Supplement: Appendix S1 — Full model equations, analysis of the effects of model variability on the dynamics of a single flip-flop model, and analysis of the differences between the model baseline and post-REM sleep deprivation conditions. (PDF) [file pone.0094481.s004.pdf]

Coupled flip-flop model for REM sleep regulation in the rat

Justin R. Dunmyre[1,4], George A. Mashour[2,3] and Victoria Booth[1,2,3,*]

[1]Department of Mathematics, University of Michigan, Ann Arbor, MI, USA
[2]Department of Anesthesiology, University of Michigan Medical School, Ann Arbor, MI, USA
[3]Neuroscience Graduate Program, University of Michigan, Ann Arbor, MI, USA
[4]Department of Mathematics, Frostburg State University, Frostburg, MD, USA
∗ E-mail: vbooth@umich.edu

Appendix S1

Coupled flip-flop model: Equations

In the coupled flip-flop model, firing rates (in Hz) of the wake-promoting, sleep-promoting, REM-on and REM-off neuronal populations, fW, fS, fR^{on}, and fR^{off}, respectively, are governed by the following equations:

$$\begin{aligned}
fW' &= (W_\infty(g_{S,W} \cdot cS + \delta(t)) - fW)/\tau_W, && \text{(S1)}\\
fS' &= (S_\infty(g_{W,S} \cdot cW) - fS)/\tau_S, && \text{(S2)}\\
fR^{on\prime} &= (R^{on}_\infty(g_{R^{off},R^{on}} \cdot cR^{off}) - fR^{on})/\tau_{R^{on}}, && \text{(S3)}\\
fR^{off\prime} &= (R^{off}_\infty(g_{R^{on},R^{off}} \cdot cR^{on}) - fR^{off})/\tau_{R^{off}}, && \text{(S4)}
\end{aligned}$$

where $'$ indicates differentiation with respect to time (in s). Concentration levels of neurotransmitters expressed by each population, $cW, cS, cR^{on}, cR^{off}$ (dimensionless with values between 0 and 1), are described by the following equations:

$$\begin{aligned}
cW' &= (\xi_W \cdot cW_\infty(fW) - cW)/\tau_{cW}, && \text{(S5)}\\
cS' &= (\xi_S \cdot cS_\infty(fS) - cS)/\tau_{cS}, && \text{(S6)}\\
cR^{on\prime} &= (\xi_{R^{on}} \cdot cR^{on}_\infty(fR^{on}) - cR^{on})/\tau_{cR^{on}}, && \text{(S7)}\\
cR^{off\prime} &= (\xi_{R^{off}} \cdot cR^{off}_\infty(fR^{off}) - cR^{off})/\tau_{cR^{off}}. && \text{(S8)}
\end{aligned}$$

The parameters $g_{X,Y}$ for $X, Y = W, S, R^{on}, R^{off}$ indicate the strength of the effect of the neurotransmitter expressed by population X on the activity of population Y. The time constants τ_X for $X = W, S, R^{on}, R^{off}$ describe the time scale of evolution of firing rate changes in population X, and the time constants τ_{cX} describe the time scale of evolution of neurotransmitter expression by population X. Population firing rate dynamics are determined by the sigmoidal steady state firing rate functions $X_\infty(c)$ given by:

$$W_\infty(c) = \frac{1}{2}W_{max} \cdot (1 + \tanh((c - \beta_{\infty,W})/\alpha_W)), \quad \text{(S9)}$$

$$S_\infty(c) = \frac{1}{2} S_{max} \cdot (1 + \tanh((c - [k_S^2 \cdot (h - k_S^1)])/\alpha_S)), \tag{S10}$$

$$R_\infty^{on}(c) = \frac{1}{2} R_{max}^{on} \cdot (1 + \tanh((c - \beta_{\infty,R^{on}})/\alpha_{R^{on}})), \tag{S11}$$

$$R_\infty^{off}(c) = \frac{1}{2} R_{max}^{off} \cdot (1 + \tanh((c - [k_{R^{off}}^2 \cdot (stp - k_{R^{off}}^1)])/\alpha_{R^{off}})), \tag{S12}$$

where α_X dictates the slope of the sigmoidal curve and $\beta_{\infty,X}$ gives the half-activation threshold. For $X = S, R^{off}$, the half-activation threshold (in square brackets) varies with the homeostatic sleep drive h and the REM sleep homeostatic drive stp, respectively, through a linear transformation with constants k_X^1 and k_X^2. Neurotransmitter expression dynamics are determined by the saturating steady state neurotransmitter release functions $cX_\infty(f)$ for $X = W, S, R^{on}, R^{off}$ given by:

$$cX_\infty(f) = \tanh(f/\gamma_X) \tag{S13}$$

where γ_X for $X = W, S, R^{on}, R^{off}$ dictates the slope of the saturating curve.

The homeostatic sleep drive, h, increases towards a maximum value h_{max} during wake (fW above a threshold value θ_W) and decreases during sleep states ($fW \leq \theta_W$) to a minimum value h_{min} with time constants $\tau_{h,up}$ and $\tau_{h,down}$, respectively:

$$h' = \begin{cases} (h_{max} - h)/\tau_{h,up} & \text{if } fW > \theta_W \\ (h_{min} - h)/\tau_{h,down} & \text{if } fW \leq \theta_W \end{cases} \tag{S14}$$

The REM sleep homeostatic drive, stp, increases towards a maximum stp_{max} during NREM sleep states ($fW \leq \theta_W$ and $fR^{on} \leq \theta_{R^{on}}$) and decreases towards a minimum stp_{min} during REM sleep states ($fW \leq \theta_W$ and $fR^{on} > \theta_{R^{on}}$) with time constants $\tau_{stp,up}$ and $\tau_{stp,down}$, respectively. During wake ($fW > \theta_W$), stp approaches the reset value stp_r with time constant $\tau_{stp,W}$:

$$stp' = \begin{cases} (stp_r - stp)/\tau_{stp,W} & \text{if } fW > \theta_W \\ (stp_{min} - stp)/\tau_{stp,down} & \text{if } fW \leq \theta_W \text{ and } fR^{on} > \theta_{R^{on}} \\ (stp_{max} - stp)/\tau_{stp,up} & \text{if } fW \leq \theta_W \text{ and } fR^{on} \leq \theta_{R^{on}} \end{cases} \tag{S15}$$

There are five sources of noise in the model. The first four modulate neurotransmitter release by the W, S, R^{on}, and, R^{off} populations; this is accomplished by randomly updating the multiplicative scaling parameters ξ_W, ξ_S, $\xi_{R^{on}}$, and $\xi_{R^{off}}$ respectively. Each parameter is updated at independent and random times, where each time is drawn from an independent Poisson distribution. For ξ_W and ξ_S we used Poisson distributions with $\lambda = 0.02$, while we used $\lambda = 0.01$ for $\xi_{R^{on}}, \xi_{R^{off}}$. When the time arose to update ξ_W, ξ_S, $\xi_{R^{on}}$ or $\xi_{R^{off}}$, a new value was chosen from a uniform distribution between 0.5 and 1.5.

The final source of noise is the external random excitatory stimuli to the wake population, $\delta(t)$. $\delta(t)$ is updated at random times drawn from a Poisson distribution where the

λ parameter is variable. When δ is updated, it is reset to $\delta = 10$, and then it decays to 0 with timescale τ_{stim} according to:

$$\delta' \quad = -\delta/\tau_{stim} \tag{S16}$$

The time of the next δ update is calculated using $\lambda = \omega$ where ω approaches a maximum value ω_{max} during REM sleep ($fR^{on} > \theta_{R^{on}}$) and approaches a minimum value ω_{min} during all other states, with a time constant τ_ω:

$$\omega' \quad = \begin{cases} (\omega_{max} - \omega)/\tau_\omega & \text{if } fR^{on} > \theta_{R^{on}} \\ (\omega_{min} - \omega)/\tau_\omega & \text{if } fR^{on} \leq \theta_{R^{on}} \end{cases} \tag{S17}$$

Parameter values for the baseline sleep condition are listed in Table S1. The following parameters were changed for the post-REM sleep deprivation condition: $h_{max} = 1.1$, $stp_{max} = 1.3$, $stp_{min} = -0.6$, $k^2_{R^{off}} = 5.25$, $\omega_{max} = 0.005$ and $\omega_{min} = 0.0007$.

Effect of variability on the dynamics of a single flip-flop model: Analysis

Here, we describe in more detail our analysis of how physiologically motivated sources of noise, which we include in our models, perturb hysteresis loop dynamics of a single flip-flop. First, we consider the effects of variability in neurotransmitter expression governed by the randomly varying terms $\xi_X(t)$ $X = W, S$) which multiplicatively scale the steady state neurotransmitter expression functions cX_∞ ($X = W, S$). As ξ_W, ξ_S take on different values, in the interval around 1, they affect the Z-shaped fixed point curve, and thus the hysteresis loop, by changing the values of RK_W and LK_W. As shown in Fig. S1, the distance between RK_W and LK_W decreases for ξ_W and ξ_S values less than 1 and increases for values greater than 1. This effect on the width of the hysteresis loop is a direct result of decreases and increases in inhibition between populations. Specifically, variations in ξ_W have a large effect on the value of RK_W by changing the level of inhibition from the wake-promoting population to the sleep-promoting population. When this inhibition is high ($\xi_W > 1$), the transition from the wake state (fW activated/fS deactivated) to the sleep state requires higher values of h to overcome this increased inhibition and induce activation of fS. Thus, RK_W increases. Conversely, when ξ_W is lower than 1, the sleep-promoting population receives less inhibition and can activate at lower values of h. Thus, RK_W decreases. Variations in ξ_S have a greater effect on LK_W by influencing the level of inhibition from the sleep-promoting population to the wake-promoting population. Higher levels of inhibition to the wake population during the simulated sleep state (fW deactivated/fS activated) require more complete deactivation of the sleep-promoting population to allow the wake population's release. Thus, h must decrease to lower values for the sleep-to-wake transition to occur and LK_W decreases. When either ξ_W or ξ_S are very low, the knees of the Z-shaped

curve coalesce and hysteresis loop dynamics disappear. In this case, inhibition between populations is too weak to support a state where one population is fully activated and the other population is fully deactivated.

Durations of wake and sleep bouts randomly vary due to the changing shape of the hysteresis loop. Given that variations in ξ_W and ξ_S induce both lengthening and shortening of the hysteresis loop, one might expect that the distribution of bout durations takes on a generally Gaussian shape centered around the duration of the deterministic model where the hysteresis loop remains fixed. This can occur when the time scale of bout durations in the deterministic model are similar to the time scale of the variability (i.e. the average frequency of ξ_W and ξ_S random variations). However, features of the bout duration distributions change when the time scales of the variability and the deterministic dynamics are different, as in our model where we assume that the neurotransmitter variability occurs at a higher frequency than wake and sleep transitions. In this situation, model simulations show that bout distributions are more positively skewed than Gaussian, with a tail of longer durations, and that the distribution peaks are at shorter durations than that of the deterministic model (Fig. S2 A,B). The majority of bout durations are shorter than the deterministic durations due to the higher frequency of variability compared to the bout duration. Namely, if we consider the movement of the trajectory around the hysteresis loop during either a wake bout or a sleep bout, the trajectory must evolve to the right or left knee, respectively, before the bout is terminated. To obtain a long bout, ξ_W and ξ_S must remain at appropriate values that define a long distance between RK_W and LK_W, or set RK_W (LK_W) near h_{max} (h_{min}) until the trajectory reaches the knee. When variability frequency is high, ξ_W or ξ_S will most likely change values before that time, thus modulating the hysteresis loop and forcing an earlier bout termination. However, occasionally ξ_w and ξ_S variability do allow a long bout to occur as evidenced by the distribution tail of long bout durations.

As an example for the introduction of asymmetry in bout duration distributions, we note that for the parameter values we consider here, the wake bout duration distribution has a longer positive tail than the sleep bout distribution, indicating that a small number of very long wake bouts do occur. Such very long wake bouts are possible because, high values of ξ_W move RK_W to high h values that approach h_{max} (=1.4 in these simulations). Thus, it is possible that ξ_W and ξ_S could take on values where $RK_W > h_{max}$ which would result in the maintenance of the wake state with h saturated at h_{max} until the next random change of ξ_W or ξ_S, thus promoting a very long wake bout. Such a mechanism for long sleep bouts is not possible for the parameter values considered here since LK_W does not approach $h_{min} = -1.6$ for any values of ξ_W or ξ_S. In the Discussion, we provide possible physiological mechanisms that could support these model dynamics.

The second source of physiologically motivated variability we include in our model is brief, random excitatory stimuli input to the wake-promoting population, $\delta(t)$, representing external inputs from brain areas not included in the model such as sensory or cortical areas. These stimuli have qualitatively different effects on the distributions of wake and

sleep bout durations (Fig. S2 C,D). These perturbations generate a bi-modal distribution of wake bout durations, with a sharp peak at very short durations indicating the brief wake bouts induced by the stimuli, and a broader peak at durations shorter than in the deterministic model. Here, wake bouts are generally shorter than the deterministic duration since stimuli-induced transitions to wake result in the trajectory being closer to the right knee at the beginning of the wake bout. The distribution of sleep bout durations takes on a more exponential-shape because sleep bouts are interrupted at random times by either brief wake bouts or early transitions to wake. The maximum possible sleep bout duration is the deterministic duration since the stimuli to the wake population cannot extend sleep bout durations.

Differences between baseline and post-REM sleep deprivation sleep patterning: Analysis

To lengthen the REM-NREM hysteresis loop by increasing the distance between the knees of $\mathcal{S}$, we adjusted the influence of stp on fR^{off} activity by decreasing its effect on the half-activation threshold of the REM-off population with a decrease in the parameter $k^2_{R^{off}}$ (see Eq. S12). In Fig. S3, the vertical distance between the curves indicates the stp interval between the left and right knees of $\mathcal{S}$, $LK_{\mathcal{S}}$ and $RK_{\mathcal{S}}$, respectively, as a function of $k^2_{R^{off}}$. Smaller values of $k^2_{R^{off}}$ increase the distance between the knees with a larger effect on $LK_{\mathcal{S}}$ which governs the REM to NREM sleep transition.
