# Supplementary material for: Coupled Flip-Flop Model for REM Sleep Regulation in the Rat
Source: PLoS One. 2014 Apr 10;9(4):e94481. doi: 10.1371/journal.pone.0094481 (PMC3983214; doi:10.1371/journal.pone.0094481)
Supplement: Table S1 — Model parameter values for baseline sleep case. (PDF) [file pone.0094481.s005.pdf]

# Coupled flip-flop model for REM sleep regulation in the rat

**Justin R. Dunmyre[1,4], George A. Mashour[2,3] and Victoria Booth[1,2,3,*]**

[1]Department of Mathematics, University of Michigan, Ann Arbor, MI, USA
[2]Department of Anesthesiology, University of Michigan Medical School, Ann Arbor, MI, USA
[3]Neuroscience Graduate Program, University of Michigan, Ann Arbor, MI, USA
[4]Department of Mathematics, Frostburg State University, Frostburg, MD, USA
* E-mail: vbooth@umich.edu

**Table S1** Model parameter values

| | | | | | | | | | | | |
|---|---|---|---|---|---|---|---|---|---|---|---|
| $g_{S,W}$ | $-2$ | $W_{max}$ | 6.5 | $k_S^1$ | 0 | $\alpha_W$ | 0.5 | $\tau_W$ | $25s$ | $\tau_{stp,up}$ | $400s$ |
| $g_{W,S}$ | $-2$ | $S_{max}$ | 5 | $k_S^2$ | $-1.5$ | $\alpha_S$ | 0.25 | $\tau_S$ | $10s$ | $\tau_{stp,down}$ | $400s$ |
| $g_{R^{off},R^{on}}$ | $-2$ | $R^{on}_{max}$ | 5 | $k^1_{R^{off}}$ | 0.8 | $\alpha_{R^{on}}$ | 0.5 | $\tau_{R^{on}}$ | $1s$ | $\tau_{stp,W}$ | $30s$ |
| $g_{R^{on},R^{off}}$ | $-5$ | $R^{off}_{max}$ | 5 | $k^2_{R^{off}}$ | 7 | $\alpha_{R^{off}}$ | 0.5 | $\tau_{R^{off}}$ | $1s$ | $\tau_{stim}$ | $10s$ |
| | | | | | | $\beta_{\infty,W}$ | $-0.3$ | $\tau_{cW}$ | $10s$ | $\tau_\omega$ | $5s$ |
| | | $h_{max}$ | 0.6 | | | $\beta_{\infty,R^{on}}$ | $-0.5$ | $\tau_{cS}$ | $10s$ | | |
| | | $h_{min}$ | 0.2 | $\gamma_W$ | 5 | | | $\tau_{cR^{on}}$ | $10s$ | $\xi_W$ | $*$ |
| | | $stp_{max}$ | 1.2 | $\gamma_S$ | 4 | | | $\tau_{cR^{off}}$ | $10s$ | $\xi_S$ | $*$ |
| $\theta_W$ | 1.5 | $stp_{min}$ | $-0.8$ | $\gamma_{R^{on}}$ | 4 | $\omega_{max}$ | 0.01 | $\tau_{h,up}$ | $600s$ | $\xi_{R^{on}}$ | $*$ |
| $\theta_{R^{on}}$ | 1.5 | $stp_r$ | 0 | $\gamma_{R^{off}}$ | 5 | $\omega_{min}$ | 0.003 | $\tau_{h,down}$ | $700s$ | $\xi_{R^{off}}$ | $*$ |

Table S1: Model parameter values for baseline sleep case
